# Sodium–glucose cotransporter 2 inhibitors for hypertension in cardiovascular–kidney–metabolic syndrome

**DOI:** 10.1113/EP092813

**Published:** 2025-11-03

**Authors:** Chunxiang Xu, Xingyu Qiu, Nan Xu, Gensheng Zhang, Enyin Lai, Liang Zhao

**Affiliations:** ^1^ Department of Nephrology Children's Hospital Zhejiang University School of Medicine, National Clinical Research Center for Child Health Hangzhou China; ^2^ Department of Physiology School of Basic Medical Sciences Zhejiang University Hangzhou China; ^3^ Department of Physiology and Pathophysiology School of Basic Medical Sciences Henan University Kaifeng China

**Keywords:** blood pressure, cardiovascular–kidney–metabolic syndrome, SGLT2 inhibitor

## Abstract

The antihypertensive mechanism of sodium–glucose cotransporter 2 (SGLT2) inhibitors has been traditionally attributed to osmotic diuresis. However, emerging evidence reveals multifaceted mechanisms beyond diuresis, including regulation of the renin–angiotensin–aldosterone system, sympathetic nervous system suppression, ion homeostasis restoration, anti‐inflammatory/antioxidant actions, weight loss and improved vascular function. These synergistic pathways collectively reduce blood pressure and provide cardiorenal protection in cardiovascular–kidney–metabolic (CKM) syndrome patients. Elucidating these multi‐target antihypertensive effects holds critical implications for optimizing therapeutic strategies, mitigating cardiorenal risks and establishing SGLT2 inhibitors as adjunctive antihypertensive therapy in CKM syndrome patients.

## Abstract (highlights)

- **What is the topic of this review?**
  This review explores the pleiotropic mechanisms of SGLT2 inhibitors in blood pressure regulation within cardiovascular–kidney–metabolic (CKM) syndrome.
- **What advances does it highlight?**
  The antihypertensive mechanisms of SGLT2 inhibitors can help patients with CKM syndrome control their blood pressure. The multi‐target antihypertensive effect of these inhibitors makes SGLT2 inhibitors a reasonable treatment strategy for hypertension in patients with CKM syndrome.

## 1 INTRODUCTION

Sodium–glucose cotransporter 2 inhibitors (SGLT2i) represent a novel class of insulin‐independent orally administered glucose‐lowering agents that have gained significant clinical attention in recent years. By selectively inhibiting glucose reabsorption in the proximal renal tubules, SGLT2i enhance urinary glucose excretion, thereby reducing blood glucose levels. Concurrently, these agents promote urinary sodium excretion, demonstrating dual therapeutic benefits: blood pressure reduction and alleviation of cardiorenal burden (Fernández‐Fernandez et al., 2023).

Cardiovascular–kidney–metabolic (CKM) syndrome epitomizes a complex pathophysiological integration of cardiovascular, renal and metabolic systems, establishing a transformative paradigm for managing these pathophysiologically linked conditions (Ndumele et al., 2023). This syndrome's clinical significance stems from its strong association with highly prevalent chronic diseases, including type 2 diabetes mellitus (T2DM), obesity, cardiovascular disease (CVD) and chronic kidney disease (CKD), which collectively drive substantial global morbidity (Aggarwal et al., 2024). Effective CKM management necessitates therapeutic strategies capable of concurrently addressing multiple risk factors, such as achieving glycaemic control, regulating blood pressure and providing simultaneous cardiorenal protection. SGLT2i, through their multifunctional profile targeting of these interconnected pathways, have emerged as important therapeutic drugs for CKM syndrome. Consequently, this review summarizes the effects of SGLT2i on blood pressure regulation in CKM syndrome.

## 2 BLOOD PRESSURE REGULATION BY SGLT2 INHIBITORS IN CARDIOVASCULAR‐KIDNEY‐METABOLIC SYNDROME

CKM syndrome is classified into five progressive stages: Stage 0 (no risk factors) to Stage 4 (established cardiovascular disease with or without kidney failure) – the category representing the highest cardiovascular risk. Population‐based studies from both the United States and China reveal that approximately 90% of adults exhibit symptoms of CKM syndrome to varying degrees. With advancing stages of CKM syndrome, CKM patients exhibit progressively elevated blood pressure (Aggarwal et al., 2024; Wang et al., 2025). Despite the widespread use of first‐line antihypertensives, such as angiotensin‐converting enzyme inhibitors, angiotensin receptor blockers, and calcium channel blockers, blood pressure control remains suboptimal in clinical practice, highlighting the urgent need for innovative therapeutic strategies. SGLT2i were originally approved for use in T2DM, but in recent years, numerous clinical studies have shown that SGLT2i not only lower the blood glucose levels of T2DM patients but also reduce their blood pressure. Comprehensive meta‐analyses of randomized controlled trials demonstrate that in T2DM patients, SGLT2i can reduce systolic (SBP)/diastolic (DBP) blood pressure by 2.0–4.5/1.0–2.0 mmHg (Baker et al., 2014; Mazidi et al., 2017). Systematic analysis of clinical studies cited in this review demonstrates that the effects of SGLT2i on blood pressure across diverse populations, including those with T2DM, CKD, CVD and hypertension, are comprehensively summarized in Table 1. These data suggest that SGLT2i could be a potentially powerful pharmacological tool for anti‐blood pressure management. Notably, these modest blood pressure reductions correlate with significant cardiorenal protection: 14% lower cardiovascular event risk and 32% lower end‐stage kidney disease in patients with T2DM and an elevated risk of CVD or in patients with T2DM and albuminuric CKD, respectively (Neal et al., 2017; Perkovic et al., 2019). One reason for this is that hypertension is a significant risk factor for T2DM, CVD and CKD. In addition, a recent study reveals that there appear to be substantial racial/ethnic differences in the cardiorenal effects of SGLT2i in patients with T2DM, with consistent benefits observed amongst White and Asian populations and consistent lack of benefits in Black populations (Kunutsor et al., 2024).

**TABLE 1:** Summary of major SGLT2i trials with effect on blood pressure.

Trial	Year of publication	Patient population	*n*	SGLT2i	Dosing	Duration	SBP change (mmHg)	DBP change (mmHg)
Weber et al.	2016	T2DM + hypertension	449	Dapagliflozin	10 mg vs. placebo	12 weeks	SBP −4.28 (−6.54, −2.02)	—
Pfeifer et al.	2017	T2DM	2313	Canagliflozin	100 and 300 mg vs. placebo	26 weeks	100 mg SBP −4.3 300 mg SBP −5.0	100 mg DBP −2.5 300 mg DBP −2.4
Neal et al.	2017	T2DM + high CVD risk	10142	Canagliflozin	100 or 300 mg vs. placebo	Average 188.2 weeks	SBP −3.9 (−4.3, −3.6)	DBP −1.4 (−1.6, −1.2)
Perkovic et al.	2019	T2DM + albuminuric CKD	4401	Canagliflozin	100 mg vs. placebo	Median 136.2 weeks	SBP −3.3 (−3.86, −2.73)	DBP −0.95 (−1.28, −0.61)
Xie et al.	2024	T2DM patients with inadequate response to metformin	406	Bexagliflozin, dapagliflozin	Bexagliflozin (20 mg) or dapagliflozin (10 mg) + metformin vs. bexagliflozin (20 mg) or dapagliflozin (10 mg)	24 weeks	SBP −6.4 with bexagliflozin SBP −6.3 with dapagliflozin	—
Hong et al.	2025	T2DM patients with inadequate response to metformin + linagliptin (5 mg/day)	235	Dapagliflozin	Dapagliflozin/linagliptinfixed dose combination (10/5 mg/day) vs. linagliptin 5 mg + placebo	24 weeks	SBP −3.68	DBP −1.47

CKD, chronic kidney disease; CVD, cardiovascular disease; DBP, diastolic blood pressure; SBP, systolic blood pressure; T2DM, type 2 diabetes mellitus.

The antihypertensive effects of SGLT2i exhibit three distinctive characteristics. First, their blood pressure‐lowering capacity appears dose‐independent, as demonstrated in pharmacodynamic studies (Georgianos & Agarwal, 2019). Second, SGLT2i demonstrate significantly greater efficacy in hypertension prevention compared to conventional glucose‐lowering therapies, with a 22% lower incidence of new‐onset hypertension versus dipeptidyl peptidase 4 inhibitors in patients with T2DM (Suzuki et al., 2024). Notably, their antihypertensive effects are potentiated when combined with other antidiabetic agents. Coadministration with metformin achieves 6.1 mmHg greater SBP reductions than SGLT2i monotherapy, whilst combined use with linagliptin shows 5.3 mmHg additional reductions (Hong et al., 2025; Xie et al., 2024). Third, SGLT2i demonstrate synergistic antihypertensive effects when combined with conventional blood pressure‐lowering therapies. In a clinical study, adding dapagliflozin to renin–angiotensin–aldosterone system (RAAS) antagonist plus diuretic therapy achieved an additional 2.4 mmHg SBP reduction, whilst combined with RAAS antagonist plus calcium channel blocker/β‐blocker regimens yielded 5.4 mmHg reduction (Weber et al., 2016). The multiple mechanisms of SGLT2i in blood pressure regulation are detailed below.

## 3 PLEIOTROPIC MECHANISMS OF SGLT2I IN BLOOD PRESSURE REGULATION

Although the initial diuretic effect of SGLT2i, characterized by increased urinary glucose excretion and acute plasma volume contraction, contributes to early blood pressure reduction, their sustained antihypertensive actions extend beyond volume depletion. These include modulation of the RAAS, suppression of sympathetic nervous system (SNS) activity, restoration of ion homeostasis, anti‐inflammatory and antioxidant effects, weight loss, and improved vascular function. Collectively, these mechanisms mediate long‐term blood pressure regulation and confer cardiorenal protection. The key mechanisms involved are described in detail below.

### 3.1 Regulation of RAAS

The RAAS constitutes a fundamental regulator of arterial blood pressure and plays a pivotal pathophysiological role in cardiovascular and renal disorders. In a male Dahl salt‐sensitive hypertensive rat model, dapagliflozin demonstrated significant modulatory effects on RAAS, including upregulation of angiotensin type 2 receptor (AT_2_R) coupled with downregulation of angiotensin‐converting enzyme (ACE) and angiotensin type 1 receptor (AT_1_R) expression, thereby lowering blood pressure (Urbanek et al., 2023). Another study confirmed that continuous infusion of a loop diuretic in 10 healthy male dogs resulted in RAAS activation, causing diuretic resistance and sodium retention, thereby impairing its function in blood pressure regulation (Adin et al., 2021). Previous studies demonstrate that SGLT2i enhances cardiorenal function by upregulating apelin and ACE2 expression, thereby activating the ACE2–Ang (1–7)–Mas receptor (MasR) protective axis (Li et al., 2021). Critically, apelin acts as a dual counter‐regulatory modulator: it serves as a positive regulator of ACE2 and a negative suppressor of the Ang II/AT_1_R pathogenic pathway in hypertension and heart failure (HF) (Zhang et al., 2023). Furthermore, the apelin–angiotensin II receptor‐like 1 protein (APJ) system directly antagonizes the ACE–Ang II–AT_1_R axis, establishing a compensatory counterbalance to RAAS overactivation (Zhang et al., 2017). Therefore, the regulation of RAAS by SGLT2i is beneficial for lowering blood pressure.

### 3.2 Suppression of SNS activity

The activation of the SNS is closely related to hypertension and is regarded as a target for treating hypertension (Sorota, 2014). An increasing number of studies have shown that SGLT2i leads to a reduction in SNS activity. A clinical study demonstrated that SGLT2i reduces blood pressure without compensatory tachycardia, suggesting attenuated SNS activity (Wan et al., 2018). A case report of an elderly diabetic HF patient treated with ipragliflozin demonstrated reduced cardiac sympathetic hyperactivity via ^123^I‐metaiodobenzylguanidine cardiac‐scintigraphy after 12 months (Kiuchi et al., 2018). Another study found that SGLT2i significantly reduced the increase in tyrosine hydroxylase and noradrenaline levels in the kidneys and hearts of mice fed a high‐fat diet (Matthews et al., 2017). Further research has revealed that SGLT2i can reduce blood pressure by inhibiting the activity of the SNS. A study demonstrated that SGLT2i reduces mean arterial pressure through renal sympathetic nerve activity inhibition during simulated exercise in a spontaneously hypertensive rat model (Kim et al., 2022). SGLT2i ameliorate hypertension in neurogenic hypertensive mice by suppressing SNS activity, and demonstrate persistent inhibition of renal sympathetic nerve activity in diabetic rabbits even under unchanged blood pressure conditions (Gueguen et al., 2020; Herat et al., 2020). In addition, SGLT2i act on cardiovascular regulatory nuclei, including the rostral ventrolateral medulla (RVLM), in which SGLT2 and SGLT1 receptors are present. By hyperpolarizing RVLM neurons via SGLT2i, these agents influence the sympathetic flow of preganglionic neurons to the sympathetic nerves in the intermediate lateral nucleus of the spinal cord, thereby reducing SNS activity, collectively lowering blood pressure (Oshima et al., 2024).

### 3.3 Restoration of ion homeostasis

SGLT2i reduces blood pressure through coordinated ion channel modulation across renal and vascular systems. In the proximal tubule, SGLT2i competitively inhibit sodium–glucose cotransport whilst downregulating Na⁺/H⁺ exchanger 3 (NHE3) expression. This dual action collectively reduces sodium reabsorption by 40%, promoting natriuresis and diuresis to decrease blood volume, ultimately lowering blood pressure (Onishi et al., 2020). Concurrently in vascular smooth muscle, SGLT2i antagonize salt‐induced vasoconstriction by suppressing cytoplasmic Ca^2^⁺ elevation via transient receptor potential channel 3 (TRPC3) and sodium–calcium exchanger 1 (NCX1) modulation (Zhao et al., 2022). This reduces cytoplasmic Ca^2^⁺ levels, ultimately lowering blood pressure.

Notably, dapagliflozin induces endothelium‐independent vasodilation by activating protein kinase G, which enhances voltage‐gated potassium (Kv) channel opening probability. This triggers membrane hyperpolarization, prolonging the closure duration of L‐type calcium channels and reducing calcium influx, ultimately lowering blood pressure (Li et al., 2018).

### 3.4 Anti‐inflammatory and antioxidant actions

SGLT2i can also reduce blood pressure through anti‐inflammatory and antioxidant effects. The SGLT2i, like empagliflozin, have been shown to attenuate or ameliorate the inflammatory profile in patients with T2DM (Iannantuoni et al., 2019). Interleukin‐6 (IL‐6) drives refractory hypertension through dual renal pathological mechanisms: epithelial sodium channel (ENaC) activation in collecting ducts to promote sodium retention (Li et al., 2010), and NF‐κB‐mediated oxidative stress in proximal tubules that enhances SGLT2 activity and sodium reabsorption (Lee et al., 2007). SGLT2i disrupts this pro‐hypertensive cascade by significantly reducing plasma and urinary IL‐6 levels in T2DM patients, as demonstrated in randomized controlled trials (Dekkers et al., 2018; Koshino et al., 2022). This IL‐6 suppression attenuates both sodium retention and renal oxidative stress, thereby improving blood pressure control in refractory hypertension (Barbaro & Harrison, 2019).

SGLT2i ameliorate oxidative stress to improve endothelial function and blood pressure through interconnected pathways: by preventing endothelial nitric oxide synthase uncoupling via sirtuin 1 activation to enhance nitric oxide (NO) bioavailability (Zhou et al., 2023); suppressing pathological reactive oxygen species generation in inflamed endothelium, thereby restoring NO signaling (Uthman et al., 2019); and inhibiting cyclooxygenase‐2‐mediated vasoconstrictive pathways to improve vasodilatory capacity (Huang et al., 2023). These actions collectively reduce vascular oxidative burden, augment NO bioactivity and ultimately lower blood pressure.

### 3.5 Weight loss and improved vascular function

Visceral adiposity and elevated body weight constitute independent risk factors for hypertension (Mertens and Van Gaal, 2000). SGLT2i‐induced urinary glucose excretion promotes caloric loss, triggering adipose tissue lipolysis and subsequent body weight reduction. Clinical studies demonstrate body weight loss in T2DM patients treated with SGLT2i (Cai et al., 2018), whilst caloric restriction significantly improves essential hypertension and arterial stiffness (Nicoll & Henein, 2018). SGLT2i improves vascular function through multiple mechanisms: attenuating endothelial cell activation, inducing direct vasodilation, ameliorating endothelial dysfunction, reducing early atherogenic alterations, and decreasing arterial wall stiffness (Gaspari et al., 2017; Ramirez et al., 2019; Solini et al., 2017). These actions collectively decrease the blood pressure.

## 4 CONCLUSION

SGLT2i provides protective effects for patients with CKM syndrome through multiple mechanisms, amongst which blood pressure regulation plays a crucial role as a key mediator. SGLT2i exert their antihypertensive effects through various mechanisms, including regulation of the RAAS, inhibition of SNS activity, restoration of ion balance, anti‐inflammatory and antioxidant effects, weight loss, and improvement of vascular function. These mechanisms collectively contribute to reducing CVD mortality, delaying CKD progression and improving metabolic dysregulation. SGLT2i should therefore be considered antihypertensive agents in addition to their glycaemic and cardio‐ and nephroprotective effects. However, the antihypertensive effect alone cannot fully explain the clinical benefits of SGLT2i for patients with CKM syndrome. Therefore, more research is needed to uncover the protective mechanisms of SGLT2i. In addition to this, SGLT2i can also lower daytime and night‐time ambulatory SBP and DBP in normotensive non‐diabetic subjects (Zanchi et al., 2022). The fact that SGLT2i reduces blood pressure in non‐diabetic normotensive individuals demonstrates that their antihypertensive mechanism operates beyond pathological compensation and is dissociated from blood glucose‐lowering effects. By refining therapeutic strategies, deepening mechanistic insights into SGLT2i, and advancing their strategic combination with other pharmacological agents, we can unlock the full potential of SGLT2i in managing CKM syndrome and resistant hypertension, ultimately offering more effective and safer therapeutic options for patients.

## AUTHOR CONTRIBUTIONS

All authors contributed to the drafting, writing, revision and editing of the manuscript. All authors have read and approved the final version of this manuscript and agree to be accountable for all aspects of the work in ensuring that questions related to the accuracy or integrity of any part of the work are appropriately investigated and resolved. All persons designated as authors qualify for authorship, and all those who qualify for authorship are listed.

## CONFLICT OF INTEREST

None declared.
